# Videofluoroscopic analysis of different volumes of liquid bolus swallowing in healthy individuals: comparison between height and sex

**DOI:** 10.6061/clinics/2017(11)08

**Published:** 2017-11

**Authors:** Marcia Regina Kfouri Bernardi Regueiro, Weslania Viviane Nascimento, Luana Casari Parreira, Roberto Oliveira Dantas

**Affiliations:** I Departamento de Oftalmologia, Otorrinolaringologia, Cirurgia de Cabeca e Pescoco, Faculdade de Medicina de Ribeirao Preto, Universidade de Sao Paulo, Ribeirao Preto, SP, BR; II Departamento de Clinica Médica, Faculdade de Medicina de Ribeirao Preto, Universidade de Sao Paulo, Ribeirao Preto, SP, BR

**Keywords:** Deglutition, Deglutition Disorders, Swallowing, Dysphagia, Height, Sex

## Abstract

**OBJECTIVE:**

The volume of swallowed bolus affects the pharyngeal transit duration. The sex and corporal height of individuals may likely influence this effect. The aim of this investigation was to determine the influence of sex and corporal height on the pharyngeal transit modification produced by the swallowed bolus volume.

**METHODS:**

Forty healthy volunteers, 20 men and 20 women, including tall (10 men and 10 women, corporal height: 1.71--2.07m) and short (10 men and 10 women, corporal height: 1.52--1.70m) persons, ranging in age between 20 and 50 years, were included in the study. Videofluoroscopic evaluation of swallowing was performed with the subjects in the sitting position. Each individual swallowed three 5 mL and three 10 mL boluses of liquid barium in a random sequence. The durations of oral transit, pharyngeal transit, pharyngeal clearance, hyoid movement, upper esophageal sphincter opening and oral-pharyngeal transit were evaluated.

**RESULTS:**

In men and women, and in taller and shorter individuals, the increase of the swallowed liquid bolus volume from 5 mL to 10 mL causes a faster transit of the bolus tail from the oral-pharyngeal transition to the upper esophageal sphincter and an increase in the duration of the upper esophageal sphincter opening, with similar alteration in men and women and in taller and shorter individuals.

**CONCLUSION:**

An increase in the swallowed liquid bolus volume from 5 mL to 10 mL causes a faster pharyngeal bolus transit and a longer bolus transit through the upper esophageal sphincter, with similar alterations in men and women and in shorter and taller individuals.

## INTRODUCTION

The volume and consistency of the swallowed bolus have an influence on the oral and pharyngeal phases of swallowing 1-5, with an increase in upper esophageal sphincter (UES) opening duration 1,3,4 and longer pharyngeal transit 3. Several studies have demonstrated that sex can be a factor in influencing the phases of swallowing 5,6, with women having longer oral-pharyngeal transit durations than men 6-10. However, several investigations did not find that sex influences the timing of swallowing events 3,11, suggesting that the observed differences are likely caused by different heights of individuals, although previous results have shown a week correlation between height and some swallowing events 3.

Superior hyoid displacement is greater in taller individuals than in shorter individuals 12. Height and sex have an influence on the size of the hypopharynx and the larynx, with an independent and interacting effect on the morphology of the pharynx and the larynx 13. In women the pharynx air column is surrounded by smaller structures, and there are no differences related to pharyngeal air column measurements 14. The pharyngeal cross-sectional area measured with individuals in the sitting position is greater in men than in women 15. These anatomical differences may influence the modifications caused by the swallowed bolus volume. The differences in swallowing timing in men and women, and with the swallowed bolus volume, may be important in patients with dysphagia. Some patients may not be able to swallow higher volumes because of their incapacity to change the swallow dynamics and to increase the oral and pharyngeal capacity with the increase in bolus volume.

Considering the hypothesis that the swallows modification caused by a 5 mL to 10 mL increase in liquid bolus volume may be different in men and women and in shorter and taller individuals, the objective of this investigation was to evaluate the modification of the oral and pharyngeal transit durations after swallows of 5mL and 10mL in men and women with different body heights.

## MATERIAL AND METHODS

Evaluation of the duration of the oral and pharyngeal phases of swallowing was performed using videofluoroscopy in 40 healthy individuals, 20 men and 20 women, ranging in age between 20 and 50 years (mean age 31.5 years) (Table 1). The height of the taller group (n=20) ranged between 1.71m and 2.07 m (mean, 1.81 m; 10 men and 10 women) and the height of the shorter group ranged between 1.52 m and 1.70 m (mean, 1.62 m; 10 men and 10 women). All volunteers had no symptoms; had no digestive, neurologic or endocrine disease; and did not undergo previous surgery. The Human Research Committee of the University Hospital of Ribeirão Preto approved the investigation (protocol HCRP 1954/2010). A written informed consent was obtained from each participant and the anonymity of each volunteer was preserved.

Evaluation of swallowing was performed using the videofluoroscopy and radiologic Arcomax angiograph (model BV 300, Phillips Veenpluis, The Netherlands), which recorded 30 frames/second. The maximum duration of the tests was 60 seconds. The examination was performed with the volunteers in the lateral position seated on a chair. Non-cued swallowing of 5mL or 10mL of liquid bolus was evaluated in triplicate in a random sequence. The liquid bolus was prepared with 30mL of liquid barium sulfate (Bariogel® 100%, Laboratório Cristália, Itapira SP, Brazil) diluted in 30mL of water and was given to the individual in a plastic cup with a final consistency that was classified as level 1 (slightly thick) in the gravity flow test proposed by the International Dysphagia Diet Standardisation Initiative (IDDSI) 16. The examinations were recorded for posterior analysis frame by frame.

The durations of the movements measured during swallowing were: a) oral transit (OT): the tongue tip at incisors to the arrival of the bolus tail at the oral-pharyngeal transition; b) pharyngeal transit (PT): the bolus tail at the oral-pharyngeal transition to the bolus tail finish as it passed the upper esophageal sphincter (UES); c) pharyngeal clearance (PC): the bolus head at the oral-pharyngeal transition to the bolus tail as it finished passing the UES; d) UES opening (UESO): the time interval between the bolus head to enter the UES to the bolus tail finish to pass the UES; e) duration of hyoid movement (HM): the time interval between the onset and the end of hyoid movement; and f) oral-pharyngeal transit (OPT): the tongue tip at incisors until the bolus tail finished passing the UES 17.

Statistical analysis was performed using ProEstat Estatistics and Research (Ribeirão Preto SP, Brazil) and a linear model with mixed effects (random and fixed effects) 18. In the tables, the results are shown as the mean and 95% confidence interval (95% CI), in milliseconds (ms). The individual results are shown in the figures as the mean of the three swallows performed by each individual for each volume. A *p*≤0.05 was considered to be statistically significant.

## RESULTS

There was no effect of bolus volume on the duration of oral transit, pharyngeal clearance, hyoid movement and oral-pharyngeal transit (*p*>0.05).

**Figure 1:**
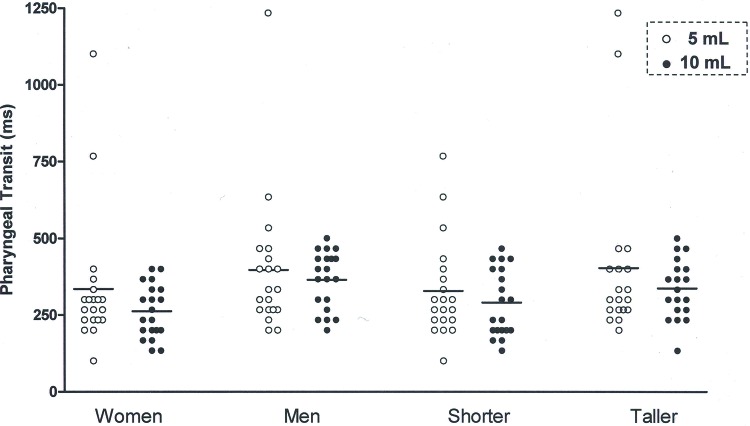
Pharyngeal transit duration, in milliseconds (ms), measured as the time between the bolus tail arriving at the oral-pharyngeal transition and the time the bolus finished passing the upper esophageal sphincter (UES). *p*≤0.05, 5 mL *vs*. 10 mL.

The pharyngeal transit duration (Figure 1) was shorter with the 10 mL bolus compared to the 5 mL bolus, for men and women (Table 2) and for taller and shorter individuals (Table 3). The differences between the mean pharyngeal transit duration for the 5 mL bolus and the mean pharyngeal transit duration for the 10 mL bolus were 35ms for men, 34ms for women, 39ms for taller subjects and 31ms for shorter subjects.

**Figure 2:**
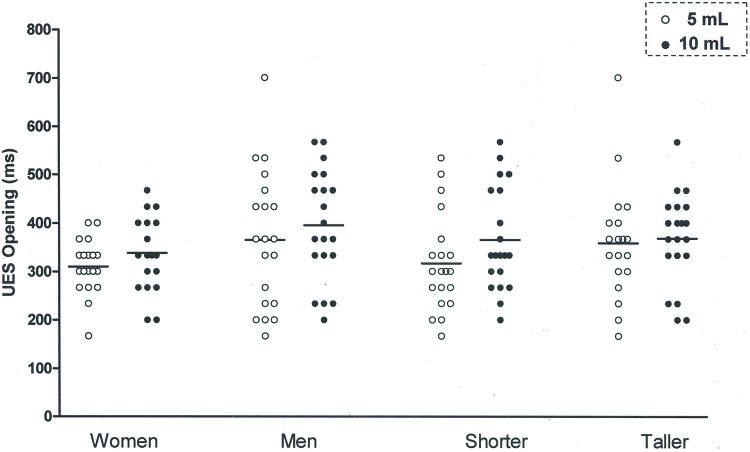
Upper esophageal sphincter (UES) opening duration, in milliseconds (ms), measured as the time between the bolus head entering the UES and the bolus tail passing the UES. *p*=0.01, 5 mL *vs*. 10 mL.

The UES opening duration (Figure 2) was longer with the 10 mL bolus than with the 5 mL bolus, for men and women (Table 2) and for taller and shorter individuals (Table 3). The differences between the mean UES opening durations for the 5mL bolus and the 10mL bolus were 36ms for men, 27ms for women, 34ms for taller subjects and 29ms for shorter individuals.

## DISCUSSION

Men have a greater oral capacity than women to accommodate a volume of liquid 19, which may be attributed to the height differences between them. Measuring the volume of the oropharyngeal cavity, the volume of the laryngeal and hypopharyngeal cavities, the length and width of the pharynx and the volume of the pyriform sinus, found higher values for men than for women 13.

This investigation demonstrated that the pharyngeal transit duration, measured from the time the bolus tail crossed the oral-pharyngeal transition to the time the bolus tail finished crossing the UES, was faster for a 10 mL liquid bolus than for a 5 mL liquid bolus. Although there are anatomic differences related to corporal height, and men and women may have some differences in swallowing dynamics, the results regarding the increase in bolus volume were similar. The faster pharyngeal bolus transit might be associated with the increase in upstream intrabolus pressure with the increase in the bolus volume 20-22, or a likely increase in the base of the tongue pressure. However, a previous investigation did not find alterations in the maximal tongue base pressure with the variation of bolus volumes 23. The explanation for the alteration of the bolus transit duration might be the UES alteration with the increase in the bolus volume, with a decrease in the minimal UES pressure with the increase in the bolus volume 23, which could facilitate bolus propulsion and transit through the pharynx.

Compared with a smaller volume, a greater bolus volume causes a longer time for the bolus to cross the UES 1,3,4,24, even with increased sphincter diameter and area when swallowing a larger volume 1,20,21. The liquid bolus length in the pharynx increases from 49.3(SE, 1.7)mm with the 5 mL bolus to 62.9(SE, 2.5)mm with the 10 mL bolus 1, and the maximum area of the UES opening, calculated as having an ellipse shape, increases from 181(SD, 59)mm^2^ with the 5 mL bolus to 195(SD, 45) mm^2^ with the 10 mL bolus 21. A 10 mL bolus arrived at the UES earlier than a 5 mL bolus and caused an early UES opening, without significantly changing the timing of the end of the bolus transit through the sphincter 21,24. The increase in bolus length and early bolus arrival at the UES can justify the increase in the duration until the bolus crossed the UES even with a faster bolus transit through the pharynx. Thus, a longer bolus length takes a longer time to cross the UES.

A previous investigation did not describe differences in pharyngeal transit duration, pharyngeal peristaltic wave amplitude and duration, and upstream intrabolus pressure in the pharynx between the swallowed liquid bolus volumes of 5 mL and 10 mL 1. In different studies, the authors used different barium concentrations. Even small differences in barium concentrations may cause modifications in the timing of swallowing events 25,26, which might explain the different results observed in the different investigations. The new consistency classification proposed by the International Dysphagia Diet Standardisation Initiative (IDDSI) has clear terminology and definitions of consistencies of liquids and solid foods, which are important for treating patients with dysphagia and in padronization of bolus consistency used in the investigation of swallowing dynamics. The proposed method for evaluating liquid consistency is ease and cost-effectiveness, using a 10 mL syringe 16,27.

A longer duration of pharyngeal transit was demonstrated only with an increased bolus volume (17.3 mL) compared to that with a smaller bolus volume (3.5mL) 3. No difference was observed between the 5 mL volume and the 10 mL volume of the thick liquid and of honey-like consistencies 4. Our results showed that the pharyngeal transit has a shorter duration with the 10 mL bolus than with the 5 mL bolus of liquid. The different conclusions of these investigations suggested that the bolus consistency and viscosity may influence the response of the pharyngeal function to the bolus volume. For the transit through the UES, the conclusions of the investigation, which compared 5 mL and 10 mL of thick liquid and honey-thick boluses, were similar in this study 4. Men and women, taller and shorter individuals, had similar body mass index, indicating that the observed differences should not be a consequence of the weight.

This study has several limitations. A larger number of volunteers would be beneficial to clarify the differences that did not reach statistical significance. The inclusion of a bolus with paste consistency might exibit the influence of height and sex on the swallowing adaptation to the bolus volumes of different consistencies. The observation of swallowing modification with bolus volume should be considered by caregivers of patients with dysphagia. Modifying the bolus volume during eating may not be possible for some of them, because of the swallowing function limitations caused by the disease. The BMI values of the groups were very similar, and the influence of the weight in the results was not expected to occur.

In conclusion, the results suggest that the increase in bolus volume from 5 mL to 10 mL causes an increase in the bolus transit duration through the UES and a decrease in the pharyngeal bolus transit duration, without differences caused by sex and corporal height.

## AUTHOR CONTRIBUTIONS

Regueiro MR, Nascimento WV, Parreira LC and Dantas RO participated in the design of the study; collection, analysis and interpretation of the data; report writing and in making the decision to submit the study for publication.

## Figures and Tables

**Table 1:** Age, height, weight and body mass index (BMI) of healthy individuals, men (n=20), women (n=20), taller (n=20) and shorter (n= 20) subjects, included in the investigation.

	MEN		WOMEN		TALLER		SHORTER	
	Mean	Limits	Mean	Limits	Mean	Limits	Mean	Limits
Age (years)	34.1	24-50	28.9	22-50	27.8	22-40	35.2	20-50
Height (m)	1.77	1.61-2.07	1.66	1.52-1.79	1.81	1.71-2.07	1.62	1.52-1.70
Weight (kg)	78.5	59.0-104.0	59.2	48.0-77.5	76.6	48.0-104.0	61.1	48.0-81.0
BMI (kg/m^2^)	25.1	21.6-29.5	21.6	18.7-28.0	23.1	18.7-29.5	23.5	19.5-28.0

**Table 2:** Oral and pharyngeal swallowing event durations (in milliseconds) in men (n=20) and women (n=20) after swallowing 5 and 10 mL liquid boluses.

	MEN					WOMEN				
	5 mL		10 mL			5 mL		10 mL		
	Mean	95% CI	Mean	95% CI	*p*	Mean	95% CI	Mean	95% CI	*p*
OT	718	633-803	672	586-759	0.17	954	809-1100	900	785-1015	0.52
PT	403	344-461	368	314-422	0.05*	272	234-309	238	219-257	0.04*
PC	636	552-718	621	556-687	0.76	448	425-472	465	433-496	0.37
UESO	355	323-386	391	359-423	0.01*	290	274-306	317	299-335	0.01*
HM	979	877-1082	894	793-994	0.44	811	706-916	862	777-946	0.27
OPT	1116	1016-1213	1042	945-1139	0.26	1223	1073-1373	1152	1015-1288	0.27

OT – oral transit; PT – pharyngeal transit; PC – pharyngeal clearance; UESO – upper esophageal sphincter opening; HM – hyoid movement; OPT – oral-pharyngeal transit.

* *p*≤0.05, 5 mL *vs*. 10 mL.

**Table 3:** Oral and pharyngeal swallowing event durations (in milliseconds) in taller (n=20) and shorter (n=20) subjects after swallowing 5 and 10 mL liquid boluses.

	TALLER					SHORTER				
	5 mL		10 mL			5 mL		10 mL		
	Mean	95% CI	Mean	95% CI	*p*	Mean	95% CI	Mean	95% CI	*p*
OT	914	758-1070	857	730-984	0.38	758	686-830	716	641-789	0.26
PT	368	304-432	329	275-385	0.05*	307	273-340	276	249-304	0.04*
PC	562	487-637	564	502-626	0.52	522	467-577	522	475-569	0.58
UESO	329	303-354	363	332-393	0.01*	316	289-342	345	321-370	0.01*
HM	867	738-996	902	820-985	0.87	923	847-1000	853	751-955	0.08
OPT	1260	1103-1417	1169	1038-1301	0.17	1079	945-1164	1024	921-1127	0.40

OT – oral transit; PT – pharyngeal transit; PC – pharyngeal clearance; UESO – upper esophageal sphincter opening; HM – hyoid movement; OPT – oral-pharyngeal transit.

* *p*≤0.05, 5 mL *vs*. 10 mL.
